# Supplementary material for: Machine learning driven biomarker selection for medical diagnosis
Source: PLoS One. 2025 Jun 11;20(6):e0322620. doi: 10.1371/journal.pone.0322620 (PMC12157214; doi:10.1371/journal.pone.0322620)
Supplement: S1 Table [file pone.0322620.s001.pdf]

**MLP**		**XGB, GBT**	
hidden_layer_sizes:	256, 128, 64, 32	max_depth:	2.0
activation:	relu	learning_rate:	1.0
random_state:	1.0	n_estimators:	10.0
		random_state:	0.0
**LR**		**RF**	
solver:	lbfgs	n_estimators:	10.0
