# Supplementary material for: Machine learning driven biomarker selection for medical diagnosis
Source: PLoS One. 2025 Jun 11;20(6):e0322620. doi: 10.1371/journal.pone.0322620 (PMC12157214; doi:10.1371/journal.pone.0322620)
Supplement: S2 Table [file pone.0322620.s002.pdf]

**Model**	**K**	**T**	**MW**	**MW-BH**	**CHI**	**CHI-BH**
MLP	3	0.538	0.448	0.542	0.581	0.594
XGB	3	0.518	0.440	0.504	0.613	0.614
LR	3	0.656	0.414	0.478	0.612	0.614
GBT	3	0.617	0.418	0.424	0.571	0.565
RF	3	0.607	0.410	0.497	0.558	0.573
MLP	10	0.701	0.586	0.512	0.669	0.659
XGB	10	0.616	0.635	0.456	0.701	0.681
LR	10	0.748	0.639	0.433	0.599	0.597
GBT	10	0.705	0.592	0.562	0.721	0.719
RF	10	0.670	0.597	0.478	0.649	0.694
