# Supplementary material for: Machine learning driven biomarker selection for medical diagnosis
Source: PLoS One. 2025 Jun 11;20(6):e0322620. doi: 10.1371/journal.pone.0322620 (PMC12157214; doi:10.1371/journal.pone.0322620)
Supplement: S3 Table [file pone.0322620.s003.pdf]

**Model**	**K**	**Test AUC**
MLP	3	0.397
XGB	3	0.453
LR	3	0.333
GBT	3	0.413
RF	3	0.469
MLP	10	0.545
XGB	10	0.600
LR	10	0.497
GBT	10	0.601
RF	10	0.555
