# Supplementary material for: Machine learning driven biomarker selection for medical diagnosis
Source: PLoS One. 2025 Jun 11;20(6):e0322620. doi: 10.1371/journal.pone.0322620 (PMC12157214; doi:10.1371/journal.pone.0322620)
Supplement: S4 Table [file pone.0322620.s004.pdf]

**Univaiate**	**Causal**
EBNA-LP_IgG	BFRF3_IgA
HP0371_IgG	IgA_1
HP0875_IgG	HP1029_IgA
