# Supplementary figures and images for: Machine learning driven biomarker selection for medical diagnosis
Source: PLoS One. 2025 Jun 11;20(6):e0322620. doi: 10.1371/journal.pone.0322620 (PMC12157214; doi:10.1371/journal.pone.0322620)

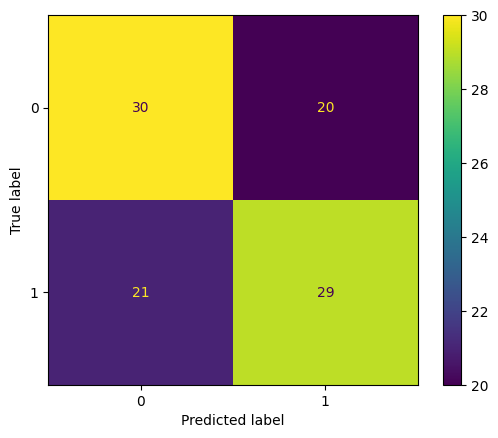

Supplement: S1 Fig [file pone.0322620.s006.tif]

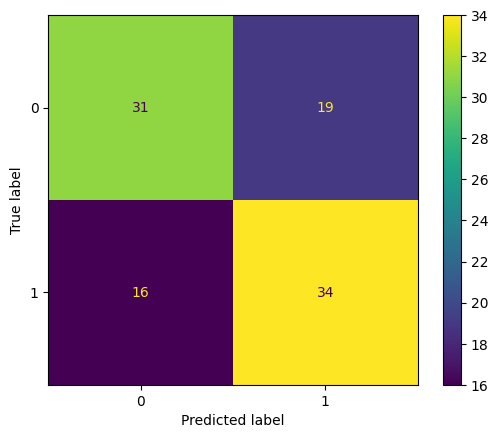

Supplement: S2 Fig [file pone.0322620.s007.tif]

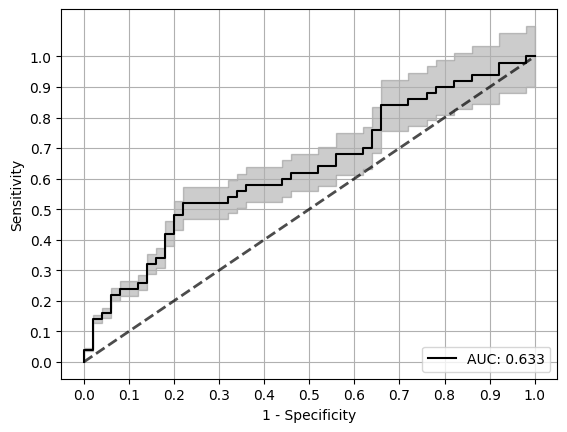

Supplement: S3 Fig [file pone.0322620.s008.tif]

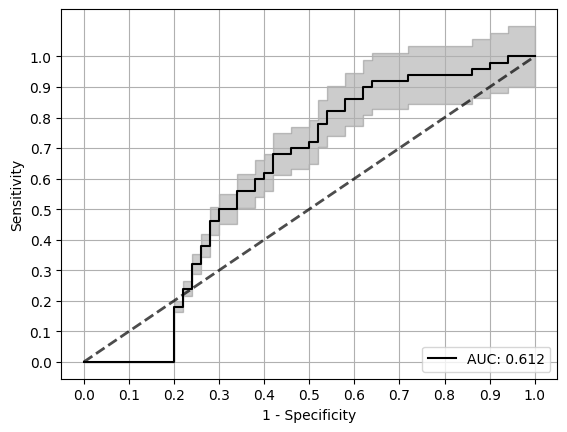

Supplement: S4 Fig [file pone.0322620.s009.tif]

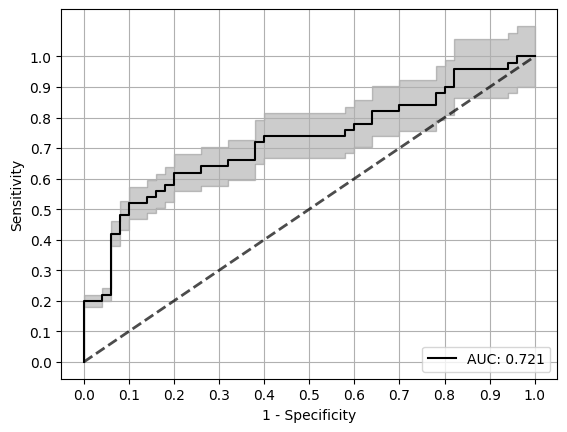

Supplement: S5 Fig [file pone.0322620.s010.tif]

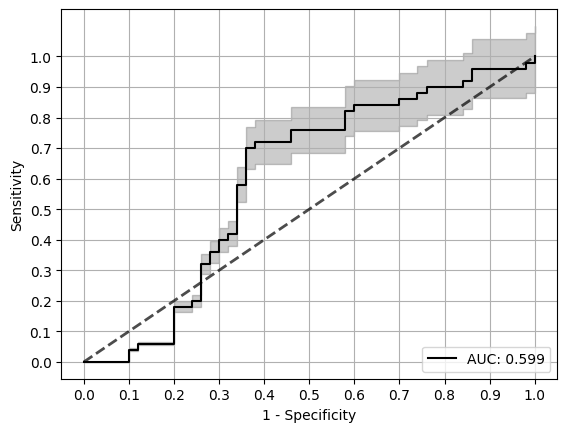

Supplement: S6 Fig [file pone.0322620.s011.tif]

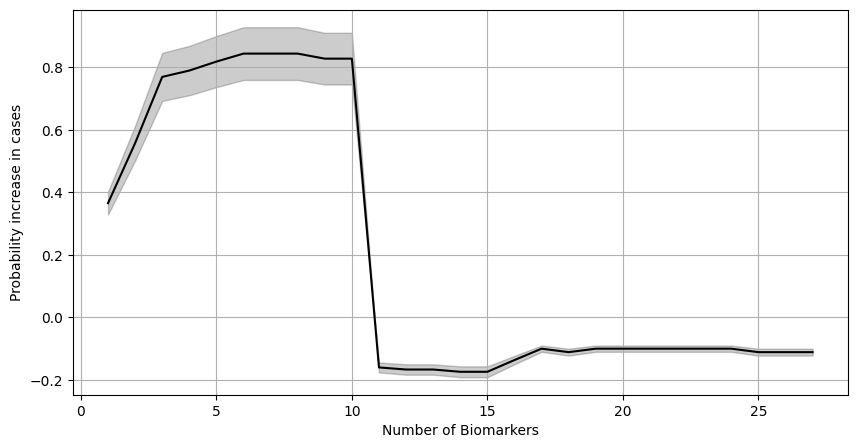

Supplement: S7 Fig [file pone.0322620.s012.tif]
