# Supplementary material for: Machine learning driven biomarker selection for medical diagnosis
Source: PLoS One. 2025 Jun 11;20(6):e0322620. doi: 10.1371/journal.pone.0322620 (PMC12157214; doi:10.1371/journal.pone.0322620)
Supplement: Attachment — Submitted filename: Response to Reviewers.docx [file pone.0322620.s013.docx]

Thank you very much for the suggestions and insightful comments into the field. We address each comment as follows:

Reviewer 1

1. We added the confusion matrix in the appendix for added performance results as figure s1. Kindly note that, AUC, ROC are more relevant in this application as they show the precision and recall tradeoff for all thresholds for the predicted probabilities and so is Sen@90 to evaluate the model that ensures in identifying most disease cases. Hence, we included those in the main paper. On the other hand, the confusion matrix reports values when a particular threshold for the predicted probabilities (=0.5) is used for classification with limited insight on the overall model performance.
2. We added a secondary analysis in the second paragraph in the discussion section assessing the increase in probability of cases based on the presence of most frequent biomarkers and also talk about future evaluation. We also talk about limitations and effect of the binarization experiments.
3. We previously included a widely applied feature selection method- univariate selection. Please note that as added in line 61-66, we chose this baseline feature selection method as it’s more applicable in this case.
4. Thank you for your comment. We added the overall workflow diagram in the second paragraph of Overview of approaches subsection in Machine Learning and Feature Selection Methods section.
5. Standard with cross validation [1,2] based ML approaches, to validate our approach, we revise two assumptions that violate most statistical tests-
   1. We have only 1 fold, that is not large enough population to apply standard statistical tests. 2. Each fold is not independent from each other as they are produced from the same dataset.
   That said, in order to communicate the significance of our results we also provide the error bars based on confidence bands on 10% error for figure 2,3,4,5.
6. We have modified the second paragraph in the related work accordingly and noted the differences in the literature to our approach. Note that medical images inherently have different attributes than protein biomarker measures and medical image recognition is not the focus of our work. We have added some more literature from line 51-66 in the second paragraph of the related works section.
7. Second paragraph in related work section is added, we also clarified that our results are not about these diagnosis in general but narrow area of disease diagnosis with the use of biomarkers with a causality measure. We clarify this and talk about how this work is positioned vis a vis the papers that the reviewer mentioned in line 53,77 in the second paragraph of the related work section and we thank the reviewer again for this insightful comment.

Reviewer 2

1. In addition to already mentioned work, we added more cancer related work as pointed out in lines 53,58,60,75.
2. We added the line 107 in Gastric Cancer Dataset section to point out that this kind of work is not explored in this particular dataset. Also, expanded our discussion in the related work to show similar work and analysis done on gastric cancer datasets.
3. The main limitation as mentioned before is for higher number of biomarkers, we add a third paragraph in the discussion section to discuss limitations about implications for overfitting, and added the fourth paragraph in the discussion section to discuss the binarized data experiments.
4. The reason for setting those threshold values is added in the last line of the first paragraph of Overview of approaches subsection in Machine Learning and Feature Selection Methods section. The justification of binarized data experiments is mentioned in the fourth paragraph in the discussion section to discuss the binarized data experiments.
5. Thanks for pointing it out, we clarify that by adding a sentence in 4^th^ line of the second paragraph of Overview of approaches subsection in Machine Learning and Feature Selection Methods section.
6. The hyperparameter details previously mentioned in appendix is moved and pointed out in the main paper in the second paragraph of the setup subsection of the results section. We also added the software packages used.
7. Thank you for your comment, we updated the legend to include asterisk and mentioned the abbreviation beforehand in the paragraph of Machine learning classification methods subsection in Machine Learning and Feature Selection Methods section.
8. We added secondary analysis to show the causal relationship of biomarkers in the second paragraph of the discussion section for this dataset. In order to compare the biomarkers with other work in the gastric cancer realm, it would have to be in a dataset where atleast all analytes with this dataset are included. We also noted that in order to fairly compare the biomarkers found, we need to conduct further clinical tests pertaining to those specific biomarkers.

References:

1. Cawley, Gavin C.; Talbot, Nicola L. C. (2010). "On Over-fitting in Model Selection and Subsequent Selection Bias in Performance Evaluation" . Journal of Machine Learning Research. 11: 2079–2107.

2. Grossman, Robert; Seni, Giovanni; Elder, John; Agarwal, Nitin; Liu, Huan (2010). "Ensemble Methods in Data Mining: Improving Accuracy Through Combining Predictions". Synthesis Lectures on Data Mining and Knowledge Discovery. 2. Morgan & Claypool: 1–126. doi:10.2200/S00240ED1V01Y200912DMK002.
