# Supplementary material for: Machine learning driven biomarker selection for medical diagnosis
Source: PLoS One. 2025 Jun 11;20(6):e0322620. doi: 10.1371/journal.pone.0322620 (PMC12157214; doi:10.1371/journal.pone.0322620)
Supplement: Attachment — Submitted filename: Response_to_Reviewers_auresp_2.docx [file pone.0322620.s014.docx]

Thank you for your insightful comments and suggestions for clarifications. We address each comment as follows:

Reviewer #2:

1. We moved the ‘Related Work’ section to a last second section, before the final section ‘Conclusion’ for clarity, so that the reader will be aware of the employed methods when reading the related work.
2. We added an example of causal metric computation on a sample dataset along with a table (table 2) in the machine learning and feature selection methods section. We also modified the description of the derivation of the causal metric in S2 Appendix to make it more clear.
3. Yes, it is 5. We fixed the typo in the Introduction section and Abstract. We updated the text to elaborate all abbreviations in the first use in page 5.
4. Thank you for your comment. We primarily used chi-square statistic based univariate feature selection as the labels in the dataset and provided by the models is discrete (binary) and in many cases we discretize the data in intermediate steps, for instance in computation of the causal measure. Also, we report results for discretized data for the univariate selection as well. However, we also experimented with statistic typically used for continuous values like both Mann-Whitney and t-tests, where it resulted in comparable or lower test AUCs. Mann-Whitney test gave lower test AUC values than all other baselines. The t-test gave test AUCs comparable to the chi-square test with a difference in performance on average is 0.01. However, for 3 biomarkers, MLP with causal method still outperforms all baselines and for 10 biomarkers, MLP with chi-square is still the best performing model as reported in the main experimental results. We include these new findings in the appendix S3, Additional tests para.
   We did try multiple hypothesis correction, more specifically, the Benjamini-Hochberg (BH) procedure. It provided superior results in limited number of settings. Although, we didn’t include it in the main paper as on average, it did not result in consistent significant improvements in the AUCs but we added them in the appendix S3, Additional tests para.
5. Thank you for pointing it out. We have results for 5% as well and we added them to the main paper as it is more standard. We added the results with 10% confidence interval in the appendix S3, Main Results with 10% confidence intervals para.
6. As mentioned in the setup subsection of the results section as well as in second paragraph of overview of approaches, we employed standard leave-one-out cross-validation (LOOCV). For a dataset of the size N, we do N iterations of train and test splits with the test size of 1 as standard with LOOCV in machine learning methods.
7. Thank you for the suggestion. We updated the main manuscript with a further elaborate figure of top 10 most frequent biomarkers over all folds for various settings of thresholds for a better view of selected biomarkers across all settings. Additionally, we also added the resulting biomarkers picked for the best performing models as a table in the appendix S3, Results of selected top K biomarkers para.
8. Yes, we did try other scaling methods like min-max scaling for the baseline of univariate selection. We add this result to the appendix S3, Experiment with min-max scaling para. This resulted in an average decline of 0.14 in the test AUCs when compared to the baseline reported in the main results section. Also, as the causal method inherently uses thresholding, scaling the data here wouldn’t significantly impact the results due to the intermediate step of thresholding (when the measurement is above the threshold, the data is considered to be 1 or 0 otherwise).
9. We updated the first para in hyperparameter study to discuss more on the effect of thresholds for binarizing the data. Generally, the threshold 1 seems to give lower test AUCs and the threshold 1.4 is the most optimal. Also, as noted in the final para’s 5^th^ line (line 243), binarized experiments perform in a similar fashion as the non-binarized ones.
